# Cost Profile of Membranes That Use Polymers of Intrinsic Microporosity (PIMs)

**DOI:** 10.3390/membranes12040433

**Published:** 2022-04-17

**Authors:** Despina A. Gkika, Volkan Filiz, Sofia Rangou, George Z. Kyzas, Athanasios C. Mitrοpoulos

**Affiliations:** 1 Department of Chemistry, International Hellenic University, 654 04 Kavala, Greece; kyzas@chem.ihu.gr (G.Z.K.); amitrop@chem.ihu.gr (A.C.M.); 2 Helmholtz-Zentrum Hereon, Institute of Membrane Research, 21502 Geesthacht, Germany; volkan.filiz@hereon.de (V.F.); sofia.rangou@hereon.de (S.R.)

**Keywords:** polymers of intrinsic microporosity, window of opportunity, total cost of ownership, cost analysis, cost profile

## Abstract

Assessing the financial impact of polymers of intrinsic microporosity, otherwise known as PIMs, at the lab scale has been impeded by the absence of a holistic approach that would envelop all related financial parameters, and most importantly any indirect costs, such as laboratory accidents that have been consistently neglected and undervalued in past assessments. To quantify the cost of PIMs in relation to the risks befalling a laboratory, an innovative cost evaluation approach was designed. This approach consists of three stages. Firstly, a two-fold “window of opportunity” (WO) theory is suggested, dividing the total cost profile into two segments, followed up by a qualitative risk analysis to establish the potential cost components. The last stage builds on a total cost of ownership model, incorporating the two types of WO. The total cost of ownership (TCO) approach was selected to ascertain the costs and construct the cost profile of PIMs, according to laboratory experimental data. This model was applied to the synthesis and physicochemical characterization processes. The quantitative analysis revealed that the most influential parameters for synthesis are accidents and energy costs. This is in contrast with the physicochemical characterization process, where the most important determinant is the energy cost.

## 1. Introduction

Microporous materials possess pores with a diameter of less than 2 nm [1]. The research on microporous materials had seen incredible progress over the past few years, with a special emphasis on the fast-emerging field of synthesizing microporous network polymers [2]. Polymers of intrinsic microporosity (PIMs) are a category of polymers that possess properly defined and interconnected micro-cavities [3]. The first generation of PIMs, known as PIM-1, first appeared in 2004 [4], followed by the Tröger’s base polymer in 2013 [5]. PIMs have a surface area between 300 and 1760 m^2^/g. Membranes that use PIMs were originally tested for the separation of phenols from aqueous solutions, displaying impressive permeability, separation degree and stability [6]. Research suggests that PIMs also exhibit great permeability and selectivity when used for gas separation [7]. Some of the advantages of PIMs include the ease of processing the solution, the fact that they come in various structures, their thermal stability and the high amount of available free volume. Furthermore, they possess a high Brunauer–Emmett–Teller (BET) surface area, and exhibit good levels of gas permeability and selectivity [8]. Over the past few years, many researchers have performed detailed evaluations of PIM materials for the purpose of membrane-based separations [8,9,10].

Partially permeable membranes are excellent candidates for filtration and desalination purposes, because they require notably less energy compared to other approaches, such as distillation or electrodialysis [11]. In general, most marketed membranes are composed of polymers, since they can be produced inexpensively. Fouling, however, translates to a poor stability that produces high long-term costs [12]. In contrast, inorganic membranes that contain ceramics or metals permit the use of heat or chemical solvents for the defouling of the membranes, thus lowering the operational costs [13]. 

Cost is the most significant parameter when considering the use of membrane technologies. There are numerous parameters influencing the synthesis and characterization cost of membranes, such as direct and indirect initial investment costs, along with any operational and maintenance costs [14]. A comprehensive economic evaluation requires that costs such as labor, equipment and facilities are considered along with the operating conditions [15]. Further costs to take into account include but are not limited to the material costs (10%), filter or membrane replacement costs (21%) and energy consumption (69%) [16]. The material cost is needed for controlling the fouling, which lowers the permeability, when foulants get attached to the membranes and feed spacers during the process [17]. Operational costs derive from the need to replace membranes and filters after their effectiveness declines [18]. Cost factors such as power density, membrane cost and lifetime, as well as the yearly loss of power density, constitute the most significant parameters affecting the unit energy cost, whereas labor and construction costs have a relatively low impact [19]. As reported by Loeb [20], the initial investment cost corresponds to a large portion of the costs, reaching about 60%, while the operational and maintenance expenses contribute far less to the total cost.

Although prior research has included an evaluation of the economic impact [21,22,23] and/or total cost [24], only a few assessments have incorporated the concept of the total cost of the synthesis and characterization costs on a lab scale. Typically, synthesis processes and recipes arise from a laboratory’s activities. As a result, synthesis recipes are commonly public knowledge that are scarcely managed. Information about them is relatively easy to accumulate, but there are no guidelines about them and their structure [25,26]. Moritz Junker found significant differences in the dimension and operating conditions between the recipes for membranes used in academia as opposed to a production scale. He pointed out that during research stages, membranes are commonly assessed under very particular circumstances, mainly intending to characterize the membrane materials [27]. 

To the best of our knowledge, there has been no attention given to incorporating the aspect of risk into the process. Academic research in a laboratory is usually expected to yield fewer risks compared to industrial processes; nevertheless, accidents occur often in a laboratory, resulting in injuries, financial damages and even death [28]. Undoubtedly, the prevention of accidents could significantly lower the total costs [29].

There is an abundance of research works trying to implement an activity-based approach to determine and calculate costs [30,31,32], which is a commonly accepted method [33] of producing a first draft of the total cost of ownership (TCO). The TCO methodology is an activity-based concept, attempting to analyze and evaluate all the relevant process costs [5]. Indirect, otherwise called overhead, costs are defined as the costs that are not directly linked to the end-product, such as occupational injuries and illnesses [34]. In such cases, potential indirect costs are typically distributed based on the time requirement of each activity. 

Against a backdrop of encouraging the potential uses of PIM membranes, this work is designed with the goal of filling the observed research gaps through an innovative model that identifies the costs of synthesis and characterization processes, through information provided in recipes using PIM membranes as derived from the laboratory’s activities, in an effort to minimize expenses. A thorough economic evaluation necessitates the calculation of indirect costs, since they can inflict a considerable financial strain [35]. This work aims to broaden past research endeavors by establishing a TCO model from a laboratory aspect, through the objective information provided by synthesis routes regarding the production of membranes and the characterization thereof from the logbooks. In this light, this work aims to formulate a cost model that takes into account a qualitative risk evaluation to determine the potential cost parameters (accident cost included) throughout the synthesis and characterization stages. Furthermore, a quantitative evaluation aims to determine the attractiveness of the resulting TCO. It is important to note that such a model has the potential to become the first step in supporting both research and financial ambitions. 

A concise description of the suggested approach, including the window of opportunity and the impact on the synthesis and characterization stages will be discussed in Section 2, whereas Section 3 demonstrates the cost profile and explains the calculation steps for the cost parameters. Moreover, it illustrates how the model can be applied for any membrane. Section 4 summarizes the key findings, providing a critical review and comparison with other works and concentrates on how this work makes a contribution to the research community and discusses recommended further work. Section 5 discusses the conclusions.

## 2. Materials and Methods

### 2.1. Window of Opportunity

The theory of window of opportunity (WO) is chiefly utilized in medicine, by establishing the ideal time to treat an illness or disease [36,37]. In business, the WO depicts the best time to make a particular investment and the best time to deal with potential competitors [38,39].

### 2.2. The Impact on Synthesis and Characterization 

**Figure 1:**
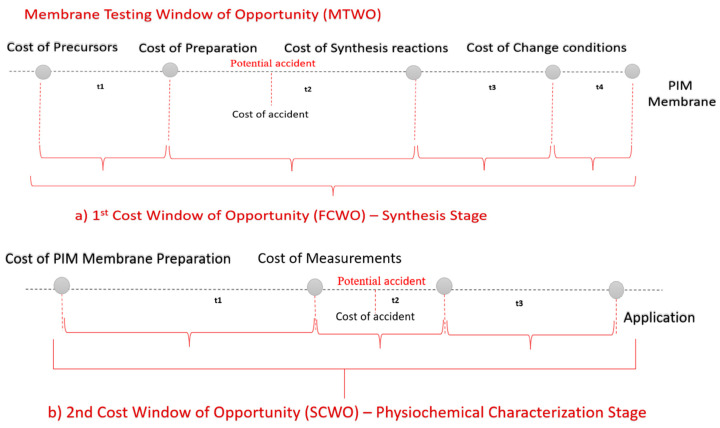
(**a**,**b**) Membrane testing cost window of opportunity in lab scale.

For the purposes of this study, the period starting right before the membrane synthesis and until its actual use, is considered as the best time to control the total cost on a lab scale. The total cost of PIM can be split between the two stages, each one corresponding to a cost window of opportunity (CWO) (Figure 1). 

Regardless of what measures are taken, or their type, each stage will result in a cost. The first stage corresponds to the synthesis stage, leading to the first CWO (FCWO). The second stage corresponds to the characterization stage, providing the second CWO (SCWO). Each cost has a different impact on the final TCO.

The FCWO consists of four segments, as shown in Figure 1a. Synthesis recipes enable the evaluation and comparison of the synthesis requirements, parameters and quality of the produced materials using a wide range of criteria. Typically, the synthesis process defines the raw materials, production steps and optimal conditions to procure the desired final product [26]. The availability of choices in recipes creates a financial potential that can support both academic and economic ambitions, thus extending the window of opportunity. Nevertheless, the majority of membranes have a low TRL indicator [40], which suggests increased levels of risks in this stage [41]. Economic losses stemming from accidents taking place may not only lead to tragic losses of life, but also cause significant social and financial losses [42]. The cost of risk during synthesis is hence higher than expected and severely limits the FCWO. It should also be noted that the estimation of the initial investment is also under-researched for low TRL technologies considering that there is little available information about the costs at this starting phase [43].

The most important part of managing the synthesis process is to comprehend the economic reasoning for intervention. The goal of analyzing the FCWO is to unveil the indirect costs occurring during the synthesis process, accidents included, in an effort to minimize any liabilities. The FCWO emphasizes the significance of checking beyond the actual synthesis process, by taking into consideration the safety-related costs as early as the design process. According to Yin, studying a synthesis recipe as a test case allows for the gathering of more reliable, concrete evidence that enables comparisons between the available choices [44]. Safety considerations should occur at the first stage of the research process [45]. Such an investment has an impact on a wide range of laboratory activities; thus, it is beneficial to evaluate the FCWO during the synthesis process.

The second cost WO (SCWO) appears between the membrane preparation and the actual application/use. The process taking place during this time is the physicochemical characterization which has different specifications than the synthesis. As a result, the main objective is to determine which stage has the highest effect on the total cost. The research of the synthesis and characterization stages aims to determine the potential lowest-cost path (FCWO and SCWO). This model can be useful in the identification of the components of the full cost profile of PIM membranes and the evaluation of our current knowledge thereof. The results could inform managers’ future decisions and assist in the appraisal of the available alternative options.

### 2.3. Experimental Section

The model and cost study were designed according to experimental data collected from previous lab projects [46].

(a) Synthesis process of PIMs

PIM-1 is one of the most prominent PIMs, and thus was used as the main source for the description of the synthesis process. McKeown [1] reported that PIMs can be synthesized through three principal approaches: the formation of dibenzodioxin [47], Troger’s base [48] and the formation of imide connections [49] between monomers. 

For the purposes of this work, PIM-1 was synthesized using the third approach [46]. All polymers were created from the various biscatechol monomers, through a reaction with a molar equivalent of 2,3,5,6- tetrafluoroterephthalonitrile (2) with an overabundance of potassium carbonate. Procedure (I) relies on the original PIM-forming reaction in DMF taking place at low temperature levels of about 65 °C over a three to four day period [12]. Procedure (II) relies on the quick synthesis of PIM-1 (one half to two hours) in dimethylacetamide (DMAc) at 150–160 °C, as explained by Guiver et al. [50].

Most chemicals were purchased from Sigma–Aldrich and required no further treatment, with a few exceptions: 2,3,5,6-tetrafluoroterephthalonitrile (2), which was donated by Lanxess and sublimated two times at 70 °C/10 3 mbar prior to use, 5,50,6,60-tetrahydroxy-3,3,30,30-tetramethyl-1,10-spirobisindane (1) and 6,60,7,70-tetrahydroxy-4,4,40,40-tetramethyl-2,20-spirobischromane (CO15) which were procured from ABCR, Germany. The co-monomers CO1, CO2, CO6, and CO19 were created in the laboratory as explained below. Potassium carbonate (Merck) was dried under vacuum at 110 °C for 12 h and milled for 10 min [46].

(b) Characterization process

**Gel permeation chromatography (GPC)**: a column combination was used at 1.0 mL/min running CHCl3 as an eluent at 30 °C temperature. About 0.4 wt.-% solutions were introduced by a 40 mL injector to the columns. Both refractive and viscosity detectors were used, with chlorobenzene serving as an internal standard. The universal calibration setting of the WINGPC software from PSS, Germany, was employed for the data assessment, using polystyrene standards for the calibration. 

**Inherent viscosity**: An Ubbelohde capillary (Nr. 501 10/1 (Schott, Mainz, Germany) was used for the measurements, maintained at a temperature of 20 °C, using a mixed solvent of CHCl3/trifluoroacetic acid (4/1 *v*/*v*) and 20 mg/cm^3^. 

**NMR spectroscopy:** The characterization of the polymers’ structure was performed through nuclear magnetic resonance spectroscopy. NMR spectra were collected using a Bruker AV300 NMR spectrometer operating at a field of 7T (300.13 MHz for 1H, 75.48 MHz for 13 °C) using a 5 mm 1H/13C TXI probe and a sample temperature of 298 K. 1H spectra were reported after applying a 10 ms 908 pulse. 13C spectra were logged using dept45, deptq135, and cpd sequences using a waltz-16 decoupling scheme. A relaxation delay was selected so that the sample was fully relaxed. 

**FT-IR spectroscopy (FT-IR):** Spectra were captured on a Bruker Equinox 55 spectrometer in ATR mode. **TGA:** Data was collected on a TG 209 F1 Iris, (Netzsch, Selb, Germany), through Argon purge. 

**Surface area measurement:** The surface area was determined using nitrogen adsorption with a Coulter 3100 instrument [46].

### 2.4. Qualitative Data

The qualitative parameters were discussed with a lab manager serving as an expert. The selected expert had to meet certain standards to ensure that they have the necessary qualifications to provide insight and support the research design and objectives. More specifically, the chosen expert is an experienced professor who has many years of experience as a lab manager, whose contributions helped to push the research forward after a set of thorough interviews (1+ h in duration), that took place in their office. The goal of these interviews was to let them unreservedly express their unbiased point of view, without utilizing predefined questions, in order to guarantee the objectivity of the designed model. This model can easily be generalized and be applied to other types of membranes.

### 2.5. Costing Method

The TCO approach is an activity-based process, heavily based on activity-based (ABC) concepts to document and assess the costs [51]. This work employed the ABC methodology since this can be easily applied in the management of a laboratory’s activities. The main focus is on accurately allocating overhead costs to end-products. Overhead costs are initially allocated to the relevant processes, then linked to cost components through the multiplication of the activity driver rate by its consumption [52]. In such cases, choosing the cost driver is significant because it may impact the accuracy of the cost evaluation. An interview with a manager can identify any causal parameter that may affect the activity’s final cost as a cost driver.

**Figure 2:**
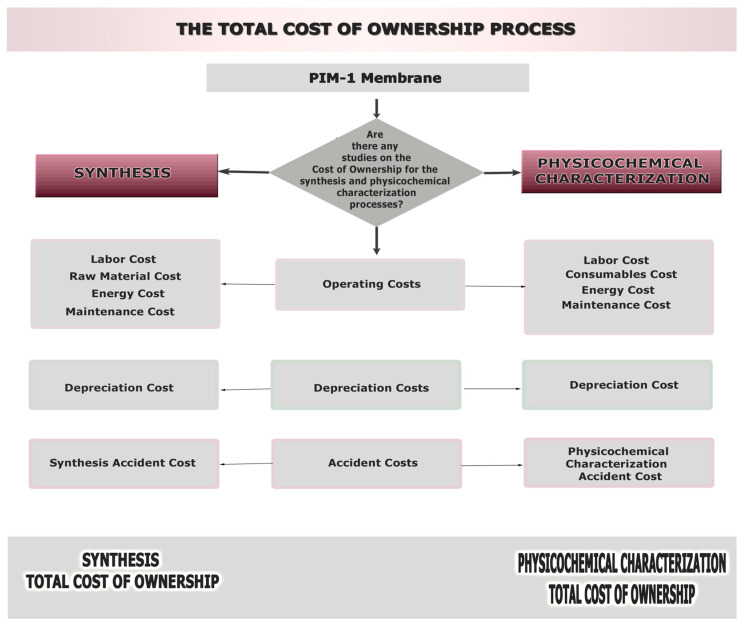
Total cost of ownership process for the synthesis and characterization phases of PIMs.

Figure 2 is a graphical representation of the TCO process, containing an analysis of the total cost of ownership parameters that will be discussed in this study.

All the relevant activities and cost factors were determined, thus establishing a TCO model for the PIMs. The activity information was provided through the expert manager interviews. Since any activity translates to a potential cost, it is crucial to be aware of what each process step requires. The main components of the cost can be divided into resources and process costs.

Labor cost [53]: The labor requirements in time for the examined synthesis and physicochemical characterization processes were retrieved from the laboratory logbooks. The synthesis labor cost can be either calculated as the total hours needed to complete the process or as the estimation of the actual labor time, considering that researchers and technicians may leave a process unattended for periods of time. It is thus worth differentiating between actual labor time and process completion time when deciding which one to incorporate in the calculation. Furthermore, it should be noted that the actual hours might also include additional activities such as a literature review for the material. This study considers the first option as more appropriate in this case. Specific assumptions have been made about the typical laboratory conditions, i.e., that one year comprises of 250 working days and one working day corresponds to 8 working hours. 

The personnel required for the synthesis of PIM-1 comprises one researcher, spending 23.75 actual person hours, while a further 2.75 h were spent during the characterization of the produced material. The average labor costs per were collected as average wages from Glassdoor [54], due to their extensive database of salary data, according to the specified position and country of residence. For each average, additional information is available about the calculation thereof (the sample size, minimum and maximum amounts, etc.). In Germany, the average hourly wage of a researcher is about EUR 28.9, while a technician is paid about EUR 25.

Energy cost: Energy use has been calculated according to the approach suggested by [55], who suggested concentrating on calculating the energy consumption of each piece of equipment used in the studied activities. This approach initially required the identification of the equipment, in an effort to calculate their nominal power, as provided in the relevant vendor technical brochures. The nominal power is higher than what is actually consumed during the process, but it may serve as a point of reference in cases where it is not possible to obtain more accurate measurements. Then, the duration of use for each equipment was documented [56]. Once all the information was collected, it was possible to calculate the actual energy consumed. The only unknown parameter remaining to be assessed was the load factor. As a result, the energy cost was calculated as the number of kWh used, multiplied by the price per kWh in euros (Germany). This price was the average for the first half of 2021, based on EU data. 

Maintenance cost: The equipment will often require repairs and replacements, which take place during maintenance [53]. Such costs include replacements, as well as the costs incurred from employee activities during the process. Periodic full-on maintenance should take place after two years for the majority of the synthesis equipment, although there are cases in which labs will proceed to replacement instead. For the characterization process, the maintenance prices are obtained from the logs of the Helmholtz–Zentrum Hereon (Institute of Membrane Research).

Depreciation cost: Depreciated cost is defined as the cost of an asset after the depreciation amount has been subtracted. It is the part that has not been fully used yet. The annuity method is often used to facilitate calculations and can be used when the annual net operating revenue remains the same and the replacement cost of the equipment is not expected to change [53]. As a result, the final cost includes both interest and depreciation. After each year, the new equipment value will equate to the discounted present value of the annuity payments left, while the depreciation cost will equate to these values. The annuity factor allows for quick calculations when there is a known current value. This might be achieved by creating a table to locate the factor for a particular rate and number of periods. A discount rate of 3% and an expected lifetime of 10 years was taken into account for both the Bruker AV300 NMR spectrometer and the Bruker ALPHA FT-IR spectrometer. An expected lifetime of 5 years was calculated for the Netzsch TG209 F1 Iris.

Accident cost: During the synthesis process, an O-ring failure allowed ethanol to leak out of a grinding jar, which then ignited inside the mill, causing a minor explosion with minor injuries to the researcher present. After investigation, it was determined that incompatible solvents were priorly used, degrading the O-ring, which combined with a delay in scheduling a service permitted a degraded O-ring to be used during the experiment. As a result, the laboratory safety guidelines were updated and employees were re-trained and encouraged to study them carefully. The accident was categorized as one of “low severity” [57] due to the minor extent of the injury.

During characterization, the accident involved occurred due to an unforeseen exothermic decomposition reaction while a technician was present. The examined samples revealed that the phenomena started at temperatures that reached above 100 ^°^C. The accident was categorized as one of “low severity” [57] due to the minor extent of the injury for the employee, prompting an absence of a few days off work. Equipment parts were ordered and replaced. No further action was required.

### 2.6. Cost Profile

**Figure 3:**
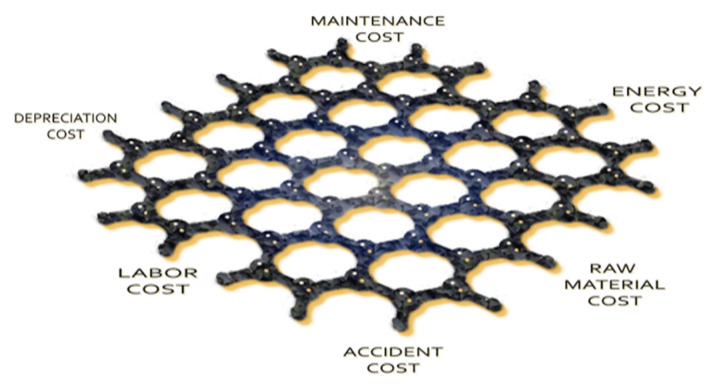
PIM membrane cost profile based on synthesis and characterization processes.

The PIM cost profile is depicted in Figure 3, presenting all the costs incurred during the synthesis and physicochemical characterization process, documenting which ones were taken into account on a lab scale. 

Cost profiling relies on the concept that any activity can be regarded as a source of cost, thus detailed information about each step, cost and activity is crucial. 

Quantitative data: Cost Model for PIM

The model comprises three tiers, namely the input, the calculations and the results, where the results depict the outcomes in a numerical or graphical way

TCO = TCOPIM = CR + CE + CL + CM + CD + CA(1)

where TCOPIM is the total cost of ownership of the synthesis and characterization process, CR—raw material cost, CE—energy cost, CL—labor cost, CM—maintenance cost, CD—depreciation of apparatus and equipment, CA—accident cost.

Ellram [33] suggested that Pareto’s law is applicable when trying to determine the cost parameters, which claims that 20% of the cost parameters constitute at least 80% of the total cost. This law is more of a generalization rather than a rule, and the percentage ratios do not have to follow those numbers precisely. The theory points out that a small number of cost parameters are frequently responsible for the majority of the total cost. The following hypothesis was thus expressed:

**Hypothesis** **1** **(H1).** *Accident cost has the highest influence on the TCO of the synthesis process and accounts for at least 80% of the total costs.*

Labor cost has the highest influence on the TCO of the characterization process and accounts for at least 80% of the total costs.

All the cost factors and the formulas used to calculate them are listed in Table 1. This table is practically the backbone of the cost model and clarifies which costs are exactly taken into account and how they were calculated.

Key: CR is the raw material cost in (EUR); *U_i_* = the price per unit for material *i* (EUR/unit); *N_i_* = the number of units for material *i* (number of units); *C_L_* is the total labor cost in (EUR); *w_i_* = the hourly wage of category *i* (EUR/hour per person); *h_i_* = the number of hours of category *i* (hours); *n_i_* = the number of employees of category *i* (number of people); *C_E_* is the cost of energy; $P_{D}$ = the nominal power of the apparatus (kW); *t* = the usage time of the apparatus (hours), where a is the load factor (0 < LF < 1); *C_M_* is the maintenance cost; *u_i_* = the price per unit for material *i* (EUR/unit); *n_i_* = the amount of units for material *i* (number of units). *w_i_* = the hourly wage of category *i* (EUR/hour per person); *h_i_* = the number of hours of category *i* (hours); *n_i_* = the number of employees of category i (number of people); *P* = payment *PV* = present value, *r* = rate per period; *n* = number of periods; CA = the annual accident cost (EUR); CMT = the cost for medical expenses (EUR); Cm * n is the medical cost × number of injured employees, no travel expenses; CRS = the salary cost for replacement staff (EUR) = Σ(*w_i_* * *h_i_* * *n_i_*) hourly wage of category *i* × hours spent × number of employees; CTR = the training cost for replacement staff (EUR) = Σ(*w_i_* * *h_i_* * *n_i_*) hourly wage of category *i* × hours spent × number of employees; CRE = the cost of replacement of equipment (EUR) = A + B + C damage to equipment + damage to infrastructure + damage to raw materials; CHB = the cost of human benefits (EUR/year) = C_1_ × n_1_ + C_2_ × n_2_C_1_; C_2_ = the cost of one light injured and serious injured worker (D/person); n1, n2 = the number of light injured and serious injured workers (# injured persons); CIB = the cost of insurance benefits (EUR/year) = P × Ip current premium*expected increase in premium (%); COC = other costs, i.e., cleaning and root cause analysis (EUR/year) = Σ(*w_i_* * *h_i_* * *n_i_*) hourly wage of category *i* × hours spent × number of employees; CA = the yearly cost of accident in euros (EUR); h_i_ = the total number of hours of category *i* (hours); H = the total number of working hours in a year (1920).

## 3. Results

**Figure 4:**
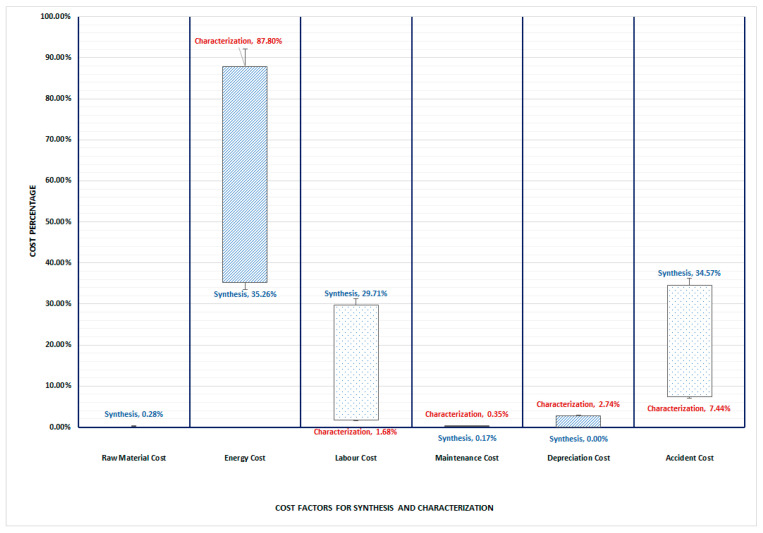
Percentage distribution of TCO cost factors per studied synthesis and characterization process.

The TCO study concentrated on determining and evaluating the costs related to the synthesis and characterization processes. The discovered cost components were categorized accordingly. To establish the cost profiles of each of the options, the cost savings were considered, including raw material costs, energy costs, labor costs, maintenance costs, depreciation costs and accident costs. Based on the developed model, the total cost of ownership for each factor is presented as a percentage distribution in Figure 4, which allows for a direct comparison of the cost distribution for the PIM, graphically.

The graph indicates that the TCO of the synthesis process is highly impacted by the energy cost (35.26%) and the accident cost (34.57%), whereas the main cost driver for characterization by far is the energy cost (87.80%) followed by the accident cost at 7.44%.

## 4. Discussion

### 4.1. Main Findings

This work identified the main variables that have an impact on the synthesis and characterization costs of a PIM membrane on a lab scale. The most significant outcomes have been summarized below:

- A new model that demonstrates how to assess the TCO for PIMs, resulting in the identification of the operating, depreciation and accident cost parameters that comprise the TCO.
- A thorough literature research and detailed expert interviews, for the identification and verification of mainly the same qualitative and quantitative factors.
- None of the identified cost factors are specific to PIMs, thus the developed model is re-usable for other types of membranes.
- It is the first time that the accident factor has been incorporated in the evaluation of a membrane’s TCO.

This work has developed and applied a TCO model which goes beyond simple cost comparisons and provides a total cost for the synthesis and characterization processes. In the case of synthesis, there is strong support for the hypothesis that the accident cost is one of the most significant factors; however, energy is slightly more impactful. Labor cost is also a crucial factor during the synthesis process, while it is practically insignificant for the characterization. Therefore, it might be interesting to further explore these factors and the differences between the two processes, since this might be indicative of the fact that the main parameter affecting the TCO, might be linked with the type of window cost of opportunity process (synthesis or characterization process).

In each work of research, it is important to determine the direct and indirect impact of such an effort and to evaluate the related costs [58]. The application of TCO concepts for PIMs enables the identification and characterization of the activities related to the synthesis and characterization thereof. The authors, on the basis of their other studies, realized that there are often costs that are unaccounted for, in which case the application of the total cost of ownership model is ideal to uncover these and take them into account for calculations regarding the synthesis and characterization processes. 

The results highlighted the need to take into account more than the price of the raw materials for the PIM, since there are other far more significant factors, such as the accident costs that constitute a critical barrier. By identifying such indirect costs, this TCO model can offer a better understanding of the cost profile of PIMs. Such knowledge is crucial when selecting between different recipes, in order to examine which options offer the lowest cost or what option could perhaps be optimized to reduce costs. The findings would thus affect the determination of which type of PIMs might be most cost effective. The TCO analysis in Section 4 illustrates how useful this kind of model could be in decision making. 

Waste is very often improperly disposed of in landfills, because of high disposal costs, without taking into account the recycling potential [59]. The waste disposal cost includes both hazardous and non-hazardous materials [60]. The increased cost stems from the need to pretreat hazardous waste before it can be appropriately disposed of in a landfill, in accordance to environmental laws and policies [61]. A solution to this problem would be to examine the potential reuse of the by-products of the process to reduce overall costs. For example, zeolite catalysts can be created from fly ash, which may reduce disposal costs and any adverse effects on the environment, among other benefits [62]. The modification of the biochar’s surface using other materials to formulate a composite that could be used as an inexpensive adsorbent for water treatment would enhance its financial value and minimize waste disposal costs, offering an alternative to expensive water treatment options such as activated carbon [63,64]. 

There is evidence to suggest that the impact of production costs has drawn very little attention in the scientific literature. It is widely accepted that the cost of production varies. Taleb et al. conceptualized a techno-economical cost model (TCM) that considered diverse production methods, material options, labor distribution, energy usage, economic factors and production goals. The model describes how the production cost per unit can be affected by contributing factors on various levels and according to the production volume [65]. Additionally, El-Gendi et al. investigated the total production cost of polyvinylchloride/cellulose acetate (PVC/CA) blend membrane sheets calculated, by considering that the raw material costs constitute approximately 50–100% of the production cost per unit, assuming it reaches an average of 75%. The production cost of a PVC/CA membrane sheet is estimated to reach about USD 32 per membrane sheet, which equates to approximately 45 USD/m^2^. The final total production cost per year was calculated to roughly USD 836k. Similarly, the cost of blended RO membrane sheets was about USD 26 per sheet, i.e., approximately 36 USD/m^2^ [66]. Viegas et al. provided a detailed cost analysis of the process as a function of the plant flow rate, which was performed pointing to total production costs of 0.21 EUR/m^3^ for a 50,000 m^3^/day plant and 20 years’ membrane lifetime [67].

There is a large body of research that argues that the cost of the membrane for the separation/ purification of substances is insignificant. For example, Pera-Titus et al. argued that contrary to traditional technologies, pervaporation (PV)-based membrane separation is simple and flexible, offers a small floor area and simplicity and possesses unique characteristics such as low cost, limited energy requirements and high efficiency. It has been deemed as an efficient and energy-saving option for cases where the separation of chemicals is not easily achieved with traditional approaches [68]. Such technology offers improved separation results and a lower energy consumption, resulting in an up to 40–60% conservation of energy [69]. In addition, Sanders et al. reported that separation based on membrane technology has gained attention owing to its reduced installation and maintenance costs, apart from the energy efficiency and other advantages such as operation and control [70]. In another study, Hinchliffe and Porter [71,72,73] performed a comparison study based on the costs of membrane separation and distillation. They defined the cost permeability and examined case studies based on the function of effective selectivity and this factor. The evaluation revealed that, in regard to the separation of membrane and gas, it is crucial to determine the optimal separation degree. When membrane separation is assessed for the cryogenic method, membranes are not effective enough; however, once the process is optimized for membrane separation, the cost is lowered to less than 60% of the cryogenic method.

### 4.2. Critical Assessment

Reflecting from a decision maker’s point of view, the qualitative analysis presented herein concentrated on the need for the segmentation and evaluation of synthesis recipes to assess their structure and the resulting information. Major publications have already started to assign a higher priority to the establishment of relevant methodologies [25,26].

Membrane manufacturers are investing in better-performing and more cost-effective technologies, and are hence considering alternatives in an effort to gradually decrease membrane costs [74]. Various assessments have been carried out to calculate the cost of membranes on a lab scale, with a noted discrepancy in the cost parameters referenced across the existing studies [75]. In industrial applications, the process design and operating conditions are led by economic concerns [76], where the main incentive is to minimize costs for specified products. For particular process design, costs can be potentially optimized by tuning the operating conditions [27]. 

This work kicked off with a conceptual design, considering raw materials, equipment cost, operating costs and labor costs [66], but in the process uncovered an additional indirect cost dimension that has not been addressed in prior works. These findings thus support a more comprehensive assessment of cost ownership. In both processes, an unexperienced researcher or technician might increase the risk of accidents; thus, there are windows of opportunity for further cost optimization during both processes. For example, proper inspection and frequent maintenance might prevent the possibility of accidents in laboratories and the costs incurred as a result.

## 5. Conclusions

This study performed an economic analysis for the synthesis and characterization of PIMs. The efficient creation of a cost profile depends on the availability of information. A TCO analysis was employed to provide quantitative estimates on the actual need and economic potential from synthesis recipes and characterization processes. Moreover, this represents the first time that the cost factor has been included in the PIM evaluation equation. Despite the clear benefits, this study uncovered a lack of information on a fundamental factor related to the processes of synthesis and characterization of PIMs on a lab scale, which is the accident cost. Although this finding may be partially idiosyncratic to the examined case studies, it highlights the importance of looking beyond the two processes, identify the window of opportunity and making use of TCO to control costs at the lab scale. 

## Figures and Tables

**Table 1:** Overview of costs and their formulas.

Cost Subcategory	Formula	Formula Number	Ref
Raw Material Cost	$\mathrm{CR}=\underset{i=1}{\sum }\left(U_{i}*N_{i}\;\right)$	(2)	[53]
Labor Cost	$C_{L}=\sum w_{i}\times h_{i}\times n_{i}$	(3)	[53]
Energy Cost	$C_{E}=P_{D}*a*t$	(4)	[55,56]
Maintenance Cost	$C_{M}=u_{i}n_{i}+w_{i}h_{i}n_{i}$	(5)	[53]
Depreciation Cost	$P=PV\times \frac{r}{1-{\left(1+r\right)}^{-n}}$	(6)	[53]
Accident cost	CA = CMT + CTR + CRS + CRE + CIB + CHB + COC	(7a)	[53]
	CR = (CA/H) * h_i_	(7b)	
